# Discrimination of Vanadium from Zinc Using Gene Profiling in Human Bronchial Epithelial Cells

**DOI:** 10.1289/ehp.7947

**Published:** 2005-06-21

**Authors:** Zhuowei Li, Jackie Stonehuerner, Robert B. Devlin, Yuh-Chin T. Huang

**Affiliations:** 1 Center for Environmental Medicine and Lung Biology, University of North Carolina, Chapel Hill, North Carolina, USA; 2 National Health and Environmental Effects Research Laboratory, U.S. Environmental Protection Agency, Research Triangle Park, North Carolina, USA

**Keywords:** cell proliferation, inflammation, interleukin-1, interleukin-8, metal, microarray, transcription

## Abstract

We hypothesized that gene expression profiling may discriminate vanadium from zinc in human bronchial epithelial cells (HBECs). RNA from HBECs exposed to vehicle, V (50 μM), or Zn (50 μM) for 4 hr (*n* = 4 paired experiments) was hybridized to Affymetrix Hu133A chips. Using one-class *t*-test with *p* < 0.01, we identified 140 and 76 genes with treatment:control ratios ≥ 2.0 or ≤ 0.5 for V and Zn, respectively. We then categorized these genes into functional pathways and compared the number of genes in each pathway between V and Zn using Fisher’s exact test. Three pathways regulating gene transcription, inflammatory response, and cell proliferation distinguished V from Zn. When genes in these three pathways were matched with the 163 genes flagged by the same statistical filtration for V:Zn ratios, 12 genes were identified. The hierarchical clustering analysis showed that these 12 genes discriminated V from Zn and consisted of two clusters. Cluster 1 genes (*ZBTB1*, *PML*, *ZNF44*, *SIX1*, *BCL6*, *ZNF450*) were down-regulated by V and involved in gene transcription, whereas cluster 2 genes (*IL8*, *IL1A*, *PTGS2*, *DTR*, *TNFAIP3*, *CXCL3*) were up-regulated and linked to inflammatory response and cell proliferation. Also, metallothionein 1 genes (*MT1F*, *MT1G*, *MT1K*) were up-regulated by Zn only. Thus, using microarray analysis, we identified a small set of genes that may be used as biomarkers for discriminating V from Zn. The novel genes and pathways identified by the microarray may help us understand the pathogenesis of health effects caused by environmental V and Zn exposure.

The advancement of microarray technology has allowed investigators to examine simultaneously changes in thousands of genes induced by environmental toxins. McDowell et al. (2000), using gene array with more than 8,000 cDNAs, found patterns of gene expression consistent with acute lung injury in nickel-treated mice. Sato et al. (1999) showed changes in genes related to cell growth and possibly carcinogenesis in rat lungs treated with diesel particles. More recently, Andrew et al. (2003) demonstrated distinct expression patterns in human lung cells exposed to low and high doses of arsenic. The capability of microarrays to provide a snapshot view of expression of a large number of genes may help us generate mechanistic hypotheses as well as identify biomarkers of exposure specific to environmental toxins. The availability of such specific genomic biomarkers may be important in determining the nature of environmental exposures.

Vanadium is present in several environmental settings, for example, during overhauling of oil-fired boilers and burning of heavy fuel in power plants. Exposures to high levels of V-rich particles produce upper and lower respiratory symptoms (Levy et al. 1984; Woodin et al. 1999, 2000). Intratracheal administration of vanadyl sulfate (VOSO_4_) and a V-rich pollutant dust, residual oil fly ash (ROFA), increased pulmonary artery pressure acutely in buffer-perfused rabbit lungs (Huang et al. 2002) and constricted isolated rat aortic rings (Cadene et al. 1997). Particulate air V concentration correlated with increases in heart rate variability index in boilermakers (Magari et al. 2002). V or ROFA altered the expression of many genes and their protein products related to acute stress (Carter et al. 1997; Gavett et al. 1997, 1999; Nadadur et al. 2000; Samet et al. 1998) and cell survival and tissue growth in cultured cells (Chen et al. 2001; Huang et al. 2000; Zhang et al. 2001).

Zinc is ubiquitous in the natural environment, including ambient air (Walsh et al. 1994). Exposure to excessive Zn (via metal fumes) is a potential hazard for industrial workers who perform welding and smelting operations. Inhalation of high concentrations of zinc oxide or zinc chloride produce respiratory epithelial cell damage, inflammation, and acute injury (Doig and Challen 1964; Evans 1945; Kuschner et al. 1995; Matarese and Matthews 1986; Nemery 1990; Pare and Sandler 1954). Treatment of lung epithelial cells *in vitro* with Zn compounds enhanced inflammatory signaling and produced cyto-toxicity and cell death (Riley et al. 2003; Samet et al. 1998, 1999).

Although V and Zn belong to different elemental classes in the periodic table, they share many biologic properties. For example, both metals are potent enhancers for phosphorylation of signaling proteins, including mitogen-activated protein kinase (Samet et al. 1998) and epidermal growth factor receptors (Wu et al. 1999), and both increase Ras activity (Wu et al. 2002) and interleukin-8 (IL8) release (Samet et al. 1998). Many of these effects may be attributed to the capability of these metals to inhibit protein tyrosine phosphatase activity (Samet et al. 1999). Both V and Zn also inhibit metabolic activity of the cells (Riley et al. 2003). V and Zn may coexist in the ambient environment after being released from different emission sources (Nriagu and Pacyna 1988). The development of a biomarker that discriminates these metals thus may help define the sources and nature of exposures. In this study we hypothesized that gene profiling may be used to discriminate V from Zn in human bronchial epithelial cells (HBECs). We sought to identify a small group of genes that may serve as biomarkers of exposure.

## Materials and Methods

### Cell culture.

Two bronchoscopists obtained bronchial epithelial cells from normal volunteers through bronchoscopic bronchial brushings following the same operational guidelines (Ghio et al. 2000; Huang et al. 2003). Subjects were informed of the procedures and potential risks, and each gave written informed consent. The protocol was approved by the University of North Carolina School of Medicine Committee on Protection of the Rights of Human Subjects and by the U.S. Environmental Protection Agency. A single experienced technician processed all brushings by following the established standard of procedures in our laboratory. The cells (passage 2 or 3) were maintained in bronchial epithelial growth medium (BEGM) (Clonetics, San Diego, CA), supplemented with bovine pituitary extract, insulin 5 μg/mL, hydrocortisone 0.5 μg/mL, gentamicin 50 μg/mL, retinoic acid 0.1 ng/mL, transferrin 10 μg/mL, triiodothyrodine 6.5 ng/mL, epinephrine 0.5 μg/mL, and human epidermal growth factor 0.5 ng/mL. Cells were judged to be 95–100% confluent at the time of metal treatment.

### Metal treatment.

Stock solutions of metals were prepared in sterile water (Baxter Healthcare Corp., Deerfield, IL) and were diluted with BEGM before experiments. Cells were grown in 100-mm diameter petri dishes and exposed to 5.5 mL of BEGM with or without 50 μM VOSO_4_ or zinc sulfate (ZnSO_4_) (Johnson Matthey Corp., Ward Hill, MA) for 4 hr.

### Purification and hybridization of RNA.

Total cellular RNA was extracted from HBECs with Trizol reagent (GIBCO BRL Life Technologies, Gaithersburg, MD) and further purified with phenol/chloroform. The RNA integrity was assessed with an Agilent 2100 bioanalyzer (Agilent Technologies, Inc., Palo Alto, CA). The 260:280-nm ratios for all RNAs were > 1.9. The RNA hybridization to the U133A GeneChip oligonucleotide microarray (Affymetrix, Inc., Santa Clara CA) was performed by Expression Analysis Inc. (Durham, NC). Affymetrix Hu133A 2.0 gene chips were used for the study. The chip contained probes for 14,500 human genes. Target was prepared and hybridized according to the Affymetrix technical manual (Affymetrix, Inc. 2004a). Total RNA (10 μg) was converted into cDNA using reverse transcriptase (Invitrogen Corp., Carlsbad, CA) and a modified oligo(dT)24 primer that contains T7 promoter sequences (GenSet Corp., San Diego, CA). After first-strand synthesis, residual RNA was degraded by the addition of RNaseH and a double-stranded cDNA molecule was generated using DNA polymerase I and DNA ligase. The cDNA was then purified and concentrated using a phenol:chloroform extraction followed by ethanol precipitation. The cDNA products were incubated with T7 RNA polymerase, and biotinylated ribonucleotides using an *in vitro* transcription kit (Enzo Diagnostics Inc., New York, NY). Half the cRNA products were purified using an RNeasy column (Qiagen Inc., Valencia, CA) and quantified with a spectrophotometer. The cRNA target (20 μg) was incubated at 94°C for 35 min in fragmentation buffer (Tris, magnesium acetate, potassium acetate). The fragmented cRNA was diluted in hybridization buffer (2-morpholinoethanesulfonic acid, NaCl, EDTA, Tween 20, herring sperm DNA, acetylated bovine serum albumin) containing biotin-labeled oligoB2 and eukaryotic hybridization controls (Affymetrix). The hybridization cocktail was denatured at 99°C for 5 min, incubated at 45°C for 5 min, and then injected into a GeneChip cartridge. The GeneChip array was incubated at 42°C for at least 16 hr in a rotating oven at 60 rpm. GeneChips were washed with a series of non-stringent (25°C) and stringent (50°C) solutions containing variable amounts of 2-morpholinoethanesulfonic acid, Tween 20, and SSPE (3 M NaCl, 0.2 M, NaH_2_PO_4_, 0.02 M EDTA). The microarrays were then stained with streptavidin phycoerythrin, and the fluorescent signal was amplified using a biotinylated antibody solution. Fluorescent images were detected in a GeneChip Scanner 3000 (Affymetrix), and expression data were extracted using the default settings in the MicroArray Suite 5.0 software (Affymetrix). All GeneChips were scaled to a median intensity setting of 500. Four independent sets of experiments were performed on HBECs obtained from four different individuals. Each set consisted of control (vehicle), VOSO_4_, and ZnSO_4_.

### Quantitative polymerase chain reaction.

Quantitative polymerase chain reaction (Q-PCR) was performed for selected genes to validate microarray results. HBECs were lysed in guanidine isothiocyanate (GITC) buffer [4 M GITC (Boehringer Mannheim, Indianapolis, IN), 25 mM sodium citrate (pH 7.0), 0.5% sarkosyl, and 0.1 M DTT], and RNA was pelleted at 80,000 rpm through a cesium chloride gradient for 2 hr at 15°C. cDNAs were synthesized from 0.4 μg of total RNA in 100 μL of a buffer containing 5 μM random hexaoligonucleotide primers (Pharmacia, Piscataway, NJ), 10 U/μL Moloney murine leukemia virus reverse transcriptase (GIBCO BRL Life Technologies), 1 U/μL RNase inhibitor (RNasin; Promega, Madison, WI), 0.5 mM dNTP (Pharmacia), 50 mM KCl, 3 mM MgCl_2_, and 10 mM Tris-HCl (pH 9.3). After 1 hr of incubation at 39°C, the reverse transcriptase was heat inactivated at 94°C for 4 min.

Q-PCR of specimen cDNA and standard cDNA was performed using TaqMan master mix (Perkin Elmer, Foster City, CA), 1.25 μM probe, 3 μM forward primer, and 3 μM reverse primer in a 50-μL volume. The probe, which contains both a fluorescence reporter dye at the 5′-end (6-carboxyfluorescein, 6-FAM: maximum emission wavelength = 518 nm) and a quencher dye at the 3′-end (6-carboxytetra-methyl rhodamine, TAMRA: maximum emission wavelength = 582 nm), is degraded by the 5′–3′ exonuclease activity of the Taq DNA polymerase, and the resulting fluorescence is detected by a laser in the sequence detector (TaqMan ABI Prism 7700 Sequence Detector System; PerkinElmer). The relative abundance of mRNA levels was determined from standard curves generated from a serially diluted standard pool of cDNA prepared from BEAS-2B cells. The relative abundance of glyceral-dehyde-3-phosphate dehydrogenase (GAPDH) mRNA was used to normalize levels of the mRNAs of interest. Six additional sets of Q-PCR experiments consisting of control (vehicle), VOSO_4_, and ZnSO_4_ were performed using HBECs from six different individuals.

### Microarray data analysis.

The microarray data were deposited in the National Center for Biotechnology Information (NCBI) Gene Expression Omnibus database (http://www.ncbi.nlm.nih.gov/geo/; accession number GSE2111). Gene expression values were background corrected and normalized globally using the default setting of the Affymetrix Microarray Suite 5.0 software, and log_2_-transformed according to the Affymetrix Statistical Algorithm Reference Guide (Affymetrix, Inc. 2004b). The log_2_ ratios of treatment (V or Zn) over control and V over Zn for all probe sets were analyzed using the one-class *t*-test against the null hypothesis of 0 (ratio = 1) using the Multiexperiment Viewer (version 3.0; The Institute of Genomic Research, Rockville, MD). A *p*-value of < 0.01 was considered statistically significant. If more than one probe set for the same gene were flagged, their ratios were averaged.

### Functional classification of genes.

Biologic processes represented by the differentially expressed genes were compiled using the GOCharts in the Database for Annotation, Visualization and Integrated Discovery (DAVID) (http://apps1.niaid.nih.gov/david/) with the coverage and specificity set at level 5 (high) and the hits threshold at 1; with the classification of the Gene Ontology Consortium (http://www.geneontology.org); and with the human gene resources from NCBI (http://www.ncbi.nlm.nih.gov). Comparison of the probe sets in the biologic processes between V and Zn was determined by the Fisher’s exact test (*p* < 0.05) (StatView 4.0; SAS Inc., Cary, NC).

## Results

### Differentially expressed genes associated with V treatment.

**Figure 1:**
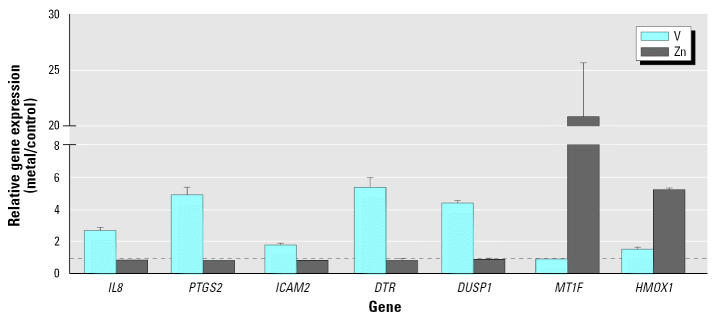
Gene expression ratios measured by Q-PCR. The expression of a gene associated with V or Zn treatment, relative to the control; *n* = 6 independent experiments in cells from six different individuals for Q-PCR. Dashed line denotes an expression ratio of 1 (no change). Data are mean ± SE.

Incubation of HBECs with VOSO_4_ at 50 μM for 4 hr showed no cytotoxicity as supported by the lack of lactate dehydrogenase (LDH) release (data not shown). There were 140 differentially expressed genes with known protein products. Seventy-six genes were up-regulated with a treatment:control ratio ≥ 2.0 (Table 1), and 64 genes were down-regulated with a treatment:control ratio ≤ 0.5 (Table 2). The expression of five up-regulated genes (*IL8*), prostaglandin-endoperoxide synthase 2 (*PTGS2*), intercellular adhesion molecule 2 (*ICAM2*), diphtheria toxin receptor (heparin-binding epidermal growth factor-like growth factor) (*DTR*), and dual specificity phosphatase 1 (*DUSP1*) was confirmed by Q-PCR in additional experiments (Figure 1). The 140 genes could be further classified functionally into 28 biologic processes containing at least three gene hits.

### Differentially expressed genes associated with Zn treatment.

Incubation of HBECs with ZnSO_4_ at 50 μM for 4 hr also showed no LDH release (data not shown). There were 76 differentially expressed genes with known protein products. Forty-three genes were up-regulated with a treatment:control ratio ≥ 2.0 (Table 3), and 33 genes were down-regulated with a treatment:control ratio ≤ 0.5 (Table 4). The up-regulation of metallothionein 1F (*MT1F*) and heme oxygenase 1 (*HMOX1*) was confirmed by Q-PCR (Figure 1). The 76 genes could be further classified into 14 biologic processes containing at least three gene hits.

### Identification of genes differentiating V from Zn.

**Figure 2:**
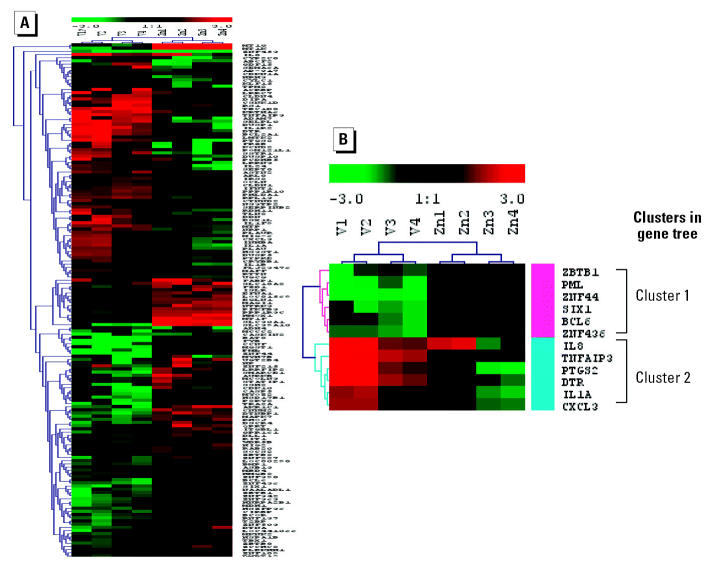
The hierarchical clustering analysis for the 163 genes that discriminated V from Zn (*A*) and the 12 genes from this list identified by additional filtration algorithms described in the text (*B*). Each row represents one single gene, and each column represents one experiment. Red areas are up-regulation, and green areas are down-regulation, relative to control. The 12 genes clearly discriminate between the V group and the Zn group. The analysis also divided the genes into two clusters. Gene names are from NCBI (http://www.ncbi.nlm.nih.gov/).

To identify genes that would discriminate V from Zn, we first analyzed V:Zn ratios using the same statistical filtration method. A total of 163 genes were identified. The results of the hierarchical clustering analysis using these genes are shown in Figure 2. We next compared biologic processes associated with V with those associated with Zn. We found that four biologic processes, regulation of transcription (24 genes), DNA-dependent transcription (22 genes), inflammatory responses (11 genes), and regulation of cell proliferation (10 genes), contained a disproportionately greater number of V-induced genes. Because all genes involved in the DNA-dependent transcription pathway were also flagged in the regulation of transcription pathway, these two processes were combined into one, designated “gene transcription.” The number of probe sets in the three biologic pathways associated with V and Zn treatment was compared using the Fisher’s exact test. The *p*-values for these three pathways, gene transcription, inflammatory response, and regulation of cell proliferation, are 0.004, 0.037, and 0.013, respectively.

We next matched genes in these three pathways with the 163 genes and identified 12 candidate genes: B-cell CLL/lymphoma 6 (*BCL6*), IL1α (*IL1A*), *IL8*, *PTGS2*, *DTR*, chemokine (C-X-C motif) ligand 3 (*CXCL3*), promyelocytic leukemia (*PML*), sine oculis homeobox homolog 1 (*Drosophila*) (*SIX1*), tumor necrosis factor (*TNF*), α -induced protein 3 (*TNFAIP3*), Zn finger and BTB domain containing 1 (*ZBTB1*), Zn finger protein 44 (KOX 7) (*ZNF44*), and Zn finger protein 450 (*ZNF450*). The hierarchical cluster analysis showed that these 12 genes clearly discriminated the V group from the Zn group (Figure 2) and could be separated into two clusters (Figure 2). Cluster 1 contained *ZBTB1*, *PML*, *ZNF44*, *SIX1*, *BCL6*, and *ZNF450* that were down-regulated by V and involved in gene transcription. Cluster 2 contained *IL8*, *IL1A*, *PTGS2*, *DTR*, *TNFAIP3*, and *CXCL3* that were up-regulated and linked primarily to inflammatory response and cell proliferation. We also noted metallothionein 1 genes were up-regulated by Zn but not by V. Zn treatment increased the expression of *MT1F* by 4.6-fold, *MT1G* by 29-fold, and *MT1K* by 81-fold. These metallothionein 1 genes constituted the third group of candidate biomarker genes.

## Discussion

In the present study we first determined the differential gene expression patterns in HBECs exposed to 50 μM of V and Zn and found 140 and 76 genes altered by V and Zn, respectively, compared with control. These genes could be classified into 28 and 14 biologic pathways, respectively, that each had at least three gene hits. Seven differentially expressed genes were validated prospectively in six additional experiments using HBECs from six different individuals. When the numbers of genes in the pathways were compared between V and Zn, three biologic processes (gene transcription, inflammatory response, and regulation of cell proliferation) contained a disproportionately greater number of V-induced genes. We then matched the genes in these three pathways with the 163 genes that differentiated V from Zn, and identified 12 candidate genes.

These 12 genes clearly discriminated the V group from the Zn group based on the hierarchical clustering analysis and could be separated into two clusters. The first cluster consisted of 6 genes (*ZBTB1*, *PML*, *ZNF44*, *SIX1*, *BCL6*, *ZNF436*) that were down-regulated by V but mildly up-regulated by Zn. All 6 genes were involved in gene transcription, and *BCL6* was also linked to inflammatory response and regulation of cell proliferation. The inhibitory effects of V on the expression of these genes have not been reported. Five of these genes encode Zn finger proteins (*ZBTB1*, *ZNF44*, *BCL6*, *ZNF436*) or proteins containing Zn-binding domains (*PML*) that play a role in DNA binding (Bray et al. 1991; Zhong et al. 2000). *SIX1* encodes a protein characterized by a divergent DNA-binding homeo-domain and an upstream *SIX* domain, which may be involved in determining DNA-binding specificity and protein–protein interactions. Mice lacking the *SIX1* gene have impaired organogenesis of skeletal muscle and kidney during embryo development (Laclef et al. 2003; Xu et al. 2003). Multiple adult tissues, including the lung, also express *SIX1* (Boucher et al. 1996), but its function is unclear. The *BCL6* gene encodes a Zn finger transcription repressor frequently associated with B-lymphocytes. Translocation and hypermutation of this gene have been detected in B-cell lymphoma (Ohno 2004). *BCL6* is also expressed in the epithelial lining of upper airways (Bajalica-Lagercrantz et al. 1998). Based on our results, *BCL6* might be involved in gene transcription, inflammatory response, and cell proliferation in airway epithelial cells. The *PML* gene encodes a Zn-binding protein in the tripartite motif (TRIM) family and is often involved in the translocation with the retinoic acid receptor-α gene associated with acute promyelocytic leukemia. High levels of PML protein are expressed in human vascular endothelial cells, epithelial cells, and macrophages (Flenghi et al. 1995).

Cluster 2 contained six genes that were up-regulated by V but down-regulated or unchanged by Zn. Four (*IL8*, *IL1A*, *PTGS2*, *CXCL3*) were related to inflammatory response, three (*IL8*, *IL1A*, *DTR*) related to regulation of cell proliferation, and two (*DTR*, *TNFAIP3*) related to gene transcription. Vanadium is known to induce IL8 in cultured bronchial epithelial cells (Carter et al. 1997; Mukherjee et al. 2004) and in the nasal fluid of workers exposed to V-rich pollutant particles (Woodin et al. 1998). Exposure to pollutant particles with high concentrations of V and Ni increased expression of PTSG2 (COX2) in nasal epithelial cells of dogs (Calderon-Garciduenas et al. 2003). Vanadium also increased the expression of DTR [heparin-binding epidermal growth factor-like growth factor (HB-EGF)] in HBECs and fibroblasts (Ingram et al. 2003; Zhang et al. 2001). The stimulatory effects of V on *IL1A* and *TNFAIP3* gene expression, however, have not been reported. *IL1A* is one of the nine genes in the IL1 gene family and is involved in various immune responses, inflammatory processes, and hematopoiesis (Arend 2002). TNFAIP3 (A20) is a Zn finger protein that is rapidly induced by TNF. It inhibits NF-κ B activation as well as TNF-mediated apoptosis (Gon et al. 2004; He and Ting 2002; Wertz et al. 2004). The *CXCL3* (*GRO-*γ ) gene is a member of a gene superfamily encoding a set of related cytokines with inflammatory and growth regulatory properties (Haskill et al. 1990). Constitutive expression of *CXCL3* has been identified in infiltrating leukocytes, bronchial epithelial cells, alveolar type II cells, and alveolar macrophages (Becker et al. 1994; Johnson et al. 1996). Several inflammatory stimuli, including IL1, TNF, lipopolysaccharide, and silica, induce the expression of CXCL3 (Becker et al. 1994; Haskill et al. 1990; Johnson et al. 1996; Rangnekar et al. 1991). Note that chemokine (C-X-C motif) ligand 1 (*CXCL1*) was also up-regulated by V (Table 1). Thus, it appears that the signaling pathways involving IL1, TNF, and chemokines activation may be novel targets for V and may play an important role in V-induced acute respiratory syndrome in boil-ermakers and power plant workers (Levy et al. 1984; Woodin et al. 2000). Up-regulation of *IL1A* and other growth-related genes (e.g., *DTR*, *FOS*, *CXCL1*, and *EDN1*) also indicates that the IL1A pathway may be also involved in clinical conditions associated with cell growth, such as fibrosis (Bonner et al. 1998, 2000).

Although not selected because they were not matched to any known pathways, several metallothionein 1 genes (*MT1F*, *MT1G*, *MT1K*) were significantly up-regulated by Zn. Metallothioneins (MT) are low-molecular-weight metal- and sulfur-rich proteins widely distributed in the organs, including the lung (Courtade et al. 1998). These intracellular proteins are thought to be involved in heavy metal detoxification and the homeostasis of essential trace metals, such as Zn and copper (Kagi 1993; Karin 1985). Exposure to zinc oxide fume increased mRNA of MTs in rat lungs (Cosma et al. 1992). Systemic administration of Zn enhanced MT levels in the liver (Conrad et al. 1997). Mice lacking MTs were more sensitive to Zn toxicity compared with wild-type mice (Park et al. 2001). In our study, in addition to increases in *MT1F* (4.6-fold), *MT1G* (29-fold), and *MT1K* (81-fold), other MTs, although not identified by our statistical filtration, also had elevated ratios: 1.36 for metallothionein 1X (*MT1X)*, 1.17 for metal-lothionein 1H (*MT1H*) and 1.21 for metal-lothionein 2A (*MT2A*). These results confirm that up-regulation of the MTs may represent early cellular defense against Zn (Irato et al. 2001; Park et al. 2001) and may be used to distinguish Zn and other heavy metals from V.

In our study, we used the one-class *t*-test with a *p*-value of < 0.01 and a ratio cutoff of ≥ 2.0 or ≤ 0.5 to identify differentially expressed genes. This statistical algorithm selected 140 genes (1.0%) from V-treated cells, 76 genes (0.5%) from Zn-treated cells, and 163 genes (1.1%) that differentiated V from Zn out of 14,500 genes in the Affymetrix Hu133A 2.0 gene chip. We are not aware of other large-scale genomic studies on V and Zn. One study reported 65 differentially expressed genes out of 1,200 genes (5.4%) associated with 4-hr 50 μM arsenic treatment in BEAS-2B cells, using a ratio cutoff of 2.0 and signal difference of 13 (Andrew et al. 2003). It is difficult to compare across the different studies, but the smaller percentage of recovery of significant genes in our study may indicate in part a more stringent filtration method. Also, the cells in our study were exposed to 50 μM VOSO_4_ and ZnSO_4_, or 14 and 18 μg of elemental V and Zn, respectively. These doses would be equivalent to working 3 hr in the environments of boilermakers and welders with the ambient V and Zn concentrations of 8 and 10 μg/m^3^, respectively (Marquart et al. 1989; Woodin et al. 2000), assuming ventilation of 10 L/min.

## Conclusion

It has been estimated that there are approximately 25,000 boilermakers and 300,000 welders nationwide. These workers can be exposed to high concentrations of V and Zn, respectively, at their workplaces. Our study compared gene expression profiles induced by V and Zn in HBECs and identified a group of 12 genes and several metallothionein 1 genes that may be used as a biomarker for V and Zn exposure, respectively. Determining the applicability of these candidate genes as biomarkers will require exposure studies enrolling a large number of subjects. The gene expression profiles provided by our study also identified potentially novel genes and pathways involved in the pathogenesis of health effects caused by environmental V and Zn exposure.

## Figures and Tables

**Table 1:** Genes up-regulated by VOSO_4_.

Gene accession no.^a^	Fold change^b^	Gene symbol^a^	Gene name^a^
Hs.624	8.04	*IL8*	interleukin 8
Hs.290873	6.67	*PPEF2*	protein phosphatase, EF hand calcium-binding domain 2
Hs.518417	5.52	*STX6*	syntaxin 6
Hs.233389	5.36	*CPVL*	carboxypeptidase, vitellogenic-like
Hs.196384	4.67	*PTGS2*	prostaglandin-endoperoxide synthase 2 (prostaglandin G/H synthase and cyclooxygenase)
Hs.248189	4.46	*KRTHA6*	keratin, hair, acidic, 6
Hs.211600	4.33	*TNFAIP3*	tumor necrosis factor, alpha-induced protein 3
Hs.477070	4.30	*CSNK1D*	casein kinase 1, delta
Hs.431460	4.24	*ICAM2*	intercellular adhesion molecule 2
Hs.44385	4.24	*SBLF*	stoned B-like factor
Hs.799	4.21	*DTR*	diphtheria toxin receptor (heparin-binding epidermal growth factor-like growth factor)
Hs.418167	4.16	*ALB*	albumin
Hs.246310	4.11	*JAM2*	junctional adhesion molecule 2
Hs.406990	4.06	*PDE4DIP*	phosphodiesterase 4D interacting protein (myomegalin)
Hs.992	4.04	*PLA2G1B*	phospholipase A2, group IB (pancreas)
Hs.496222	3.97	*ANGPTL1*	angiopoietin-like 1
Hs.65758	3.78	*ITPR3*	inositol 1,4,5-triphosphate receptor, type 3
Hs.66713	3.70	*DIPA*	hepatitis delta antigen-interacting protein A
Hs.519884	3.65	*GCNT2*	glucosaminyl (*N*-acetyl) transferase 2, I-branching enzyme
Hs.157259	3.64	*SIPA1L3*	signal-induced proliferation-associated 1-like 3
Hs.436023	3.56	*PRDM1*	PR domain containing 1, with ZNF domain
Hs.303980	3.51	*CYP11A1*	cytochrome P450, family 11, subfamily A, polypeptide 1
Hs.236646	3.49	*HOXD9*	homeo box D9
Hs.171695	3.46	*DUSP1*	dual specificity phosphatase 1
Hs.197693	3.44	*CACNG2*	calcium channel, voltage-dependent, gamma subunit 2
Hs.485910	3.34	*RARSL*	arginyl-tRNA synthetase-like
Hs.211238	3.30	*IL1F9*	interleukin 1 family, member 9
Hs.520319	3.30	*SLC22A16*	solute carrier family 22 (organic cation transporter), member 16
Hs.445555	3.22	*SERPINI2*	serine (or cysteine) proteinase inhibitor, clade I (neuroserpin), member 2
Hs.256667	3.20	*PDK2*	pyruvate dehydrogenase kinase, isoenzyme 2
Hs.248122	3.10	*GPR24*	G-protein-coupled receptor 24
Hs.511899	3.02	*EDN1*	endothelin 1
Hs.523506	2.99	*WARS2*	tryptophanyl tRNA synthetase 2 (mitochondrial)
Hs.333175	2.86	*PLA2G12B*	phospholipase A2, group XIIB
Hs.410817	2.78	*RPL13*	ribosomal protein L13
Hs.520942	2.77	*CLDN4*	claudin 4
Hs.50823	2.74	*PDCD6*	programmed cell death 6
Hs.550498	2.72	*RCE1*	RCE1 homolog, prenyl protein protease (*S. cerevisiae*)
Hs.436023	2.67	*PRDM1*	PR domain containing 1, with ZNF domain
Hs.421724	2.66	*CTSG*	cathepsin G
Hs.2250	2.63	*LIF*	leukemia inhibitory factor (cholinergic differentiation factor)
Hs.282387	2.58	*RPC32*	polymerase (RNA) III (DNA directed) (32 kDa)
Hs.525389	2.56	*ARHJ*	ras homolog gene family, member J
Hs.106019	2.54	*PPP1R10*	protein phosphatase 1, regulatory subunit 10
Hs.250281	2.52	*SLC13A3*	solute carrier family 13 (sodium-dependent dicarboxylate transporter), member 3
Hs.2128	2.48	*DUSP5*	dual-specificity phosphatase 5
Hs.89690	2.45	*CXCL3*	chemokine (C-X-C motif) ligand 3
Hs.11169	2.45	*MIG-6*	mitogen-inducible gene 6
Hs.789	2.41	*CXCL1*	chemokine (C-X-C motif) ligand 1 (melanoma growth- stimulating activity, alpha)
Hs.485004	2.37	*ZNF306*	zinc finger protein 306
Hs.534478	2.36	*DUSP21*	dual-specificity phosphatase 21
Hs.441972	2.34	*IFNT1*	interferon tau-1
Hs.503598	2.33	*JMJD2D*	jumonji domain containing 2D
Hs.546252	2.25	*EDG3*	endothelial differentiation, sphingolipid G-protein-coupled receptor, 3
Hs.85862	2.23	*PDLIM3*	PDZ and LIM domain 3
Hs.445489	2.22	*PLEKHB1*	pleckstrin homology domain containing, family B (evectins), member 1
Hs.1722	2.21	*IL1A*	interleukin 1, alpha
Hs.466871	2.21	*PLAUR*	plasminogen activator, urokinase receptor
Hs.159291	2.20	*DRP2*	dystrophin-related protein 2
Hs.303649	2.19	*CCL2*	chemokine (C-C motif) ligand 2
Hs.111944	2.19	*CYP3A7*	cytochrome P450, family 3, subfamily A, polypeptide 7
Hs.533683	2.19	*FGFR2*	fibroblast growth factor receptor 2
Hs.50550	2.19	*KBTBD10*	kelch repeat and BTB (POZ) domain containing 10
Hs.78944	2.19	*RGS2*	regulator of G-protein signaling 2, 24 kDa
Hs.190783	2.17	*HAL*	histidine ammonialyase
Hs.463059	2.17	*STAT3*	signal transducer and activator of transcription 3 (acute- phase response factor)
Hs.25647	2.16	*FOS*	v-fos FBJ murine osteosarcoma viral oncogene homolog
Hs.127022	2.14	*PTPRE*	protein tyrosine phosphatase, receptor type, E
Hs.447899	2.13	*SIGLEC8*	sialic acid-binding Ig-like lectin 8
Hs.344812	2.13	*TREX1*	three prime repair exonuclease 1
Hs.528670	2.12	*MMP25*	matrix metalloproteinase 25
Hs.514913	2.11	*SERPINB2*	serine (or cysteine) proteinase inhibitor, clade B (ovalbumin), member 2
Hs.506381	2.07	*FGD6*	FYVE, RhoGEF and PH domain containing 6
Hs.278658	2.06	*KRTHB6*	keratin, hair, basic, 6 (monilethrix)
Hs.439060	2.08	*CLDN1*	claudin 1
Hs.507348	2.05	*HS3ST1*	heparan sulfate (glucosamine) 3-*O*-sulfotransferase 1

Only genes with known protein products are shown.

a Gene annotations are from NCBI (http://www.ncbi.nlm.nih.gov).

b Fold changes are the average of four individuals.

**Table 2:** Genes down-regulated by VOSO_4_.

Gene accession no.^a^	Fold change^b^	Gene symbol^a^	Gene name^a^
Hs.441975	−11.75	*HSXIAPAF1*	XIAP-associated factor-1
Hs.370503	−8.11	*FYB*	FYN-binding protein (FYB-120/130)
Hs.76884	−7.48	*ID3*	inhibitor of DNA binding 3, dominant negative helix-loop- helix protein
Hs.520506	−7.37	*FBXO5*	F-box only protein 5
Hs.22393	−6.90	*DENR*	density-regulated protein
Hs.433060	−6.86	*ACPP*	acid phosphatase, prostate
Hs.37045	−6.77	*PTH*	parathyroid hormone
Hs.282410	−6.69	*CALM1*	calmodulin 1 (phosphorylase kinase, delta)
Hs.474251	−6.60	*SCARF2*	scavenger receptor class F, member 2
Hs.534101	−5.89	*MYO3B*	myosin IIIB
Hs.442578	−5.53	*LHX9*	LIM homeobox 9
Hs.292356	−5.29	*CYBB*	cytochrome b-245, beta polypeptide (chronic granulomatous disease)
Hs.1973	−5.21	*CCNF*	cyclin F
Hs.24684	−5.12	*ARRDC3*	arrestin domain containing 3
Hs.350756	−4.44	*STAU2*	staufen, RNA-binding protein, homolog 2 (*Drosophila*)
Hs.133892	−4.41	*TPM1*	tropomyosin 1 (alpha)
Hs.24120	−4.23	*ZNF44*	zinc finger protein 44 (KOX 7)
Hs.275243	−3.89	*S100A6*	S100 calcium-binding protein A6 (calcyclin)
Hs.526464	−3.71	*PML*	promyelocytic leukemia
Hs.522090	−3.55	*GALT*	galactose-1-phosphate uridylyltransferase
Hs.432424	−3.51	*TPP2*	tripeptidyl peptidase II
Hs.485233	−3.47	*MAPK14*	mitogen-activated protein kinase 14
Hs.434924	−3.44	*RIMS3*	regulating synaptic membrane exocytosis 3
Hs.75294	−3.38	*CRH*	corticotropin-releasing hormone
Hs.173984	−3.16	*TBX1*	T-box 1
Hs.444106	−3.11	*TOR2A*	torsin family 2, member A
Hs.254042	−3.02	*BAT1*	HLA-B associated transcript 1
Hs.75862	−2.96	*MADH4*	MAD, mothers against decapentaplegic homolog 4 (*Drosophila*)
Hs.498292	−2.89	*SDCCAG8*	serologically defined colon cancer antigen 8
Hs.1650	−2.78	*SLC26A3*	solute carrier family 26, member 3
Hs.293798	−2.69	*ZNF436*	zinc finger protein 436
Hs.397073	−2.66	*PMS2L5*	postmeiotic segregation increased 2-like 5
Hs.54416	−2.63	*SIX1*	sine oculis homeobox homolog 1 (*Drosophila*)
Hs.118513	−2.59	*MGC21621*	G-protein-coupled receptor MrgF
Hs.129634	−2.57	*CINP*	cyclin-dependent kinase 2-interacting protein
Hs.21388	−2.55	*ZDHHC21*	zinc finger, DHHC domain containing 21
Hs.131846	−2.51	*TAF6L*	TAF6-like RNA polymerase II, p300/CBP-associated factor (PCAF)-associated factor, 65 kDa
Hs.116622	−2.46	*ZFP30*	likely ortholog of mouse zinc finger protein 30
Hs.478588	−2.41	*BCL6*	B-cell CLL/lymphoma 6 (zinc finger protein 51)
Hs.47712	−2.41	*ZNF555*	zinc finger protein 555
Hs.487774	−2.41	*HNRPA2B1*	heterogeneous nuclear ribonucleoprotein A2/B1
Hs.339	−2.37	*P2RY2*	purinergic receptor P2Y, G-protein coupled, 2
Hs.501309	−2.35	*CIRBP*	cold-inducible RNA-binding protein
Hs.534040	−2.33	*KBTBD6*	kelch repeat and BTB (POZ) domain containing 6
Hs.6093	−2.27	*ARRDC4*	arrestin domain containing 4
Hs.400802	−2.27	*ZBTB1*	zinc finger and BTB domain containing 1
Hs.474799	−2.25	*PDXP*	pyridoxal (pyridoxine, vitamin B6) phosphatase
Hs.13982	−2.23	*KCTD6*	potassium channel tetramerisation domain containing 6
Hs.487561	−2.22	*ICA1*	islet cell autoantigen 1, 69 kDa
Hs.48297	−2.17	*ZNF363*	zinc finger protein 363
Hs.424926	−2.14	*TM7SF1*	transmembrane 7 superfamily member 1 (up-regulated in kidney)
Hs.101937	−2.14	*SIX2*	sine oculis homeobox homolog 2 (*Drosophila*)
Hs.409876	−2.12	*ZNF450*	zinc finger protein 450
Hs.63335	−2.12	*TERF2*	telomeric repeat binding factor 2
Hs.105633	−2.12	*WINS1*	WINS1 protein with *Drosophila* Lines (Lin) homologous domain
Hs.142167	−2.11	*HSZFP36*	ZFP-36 for a zinc finger protein
Hs.186424	−2.09	*BCOR*	BCL6 co-repressor
Hs.518438	−2.08	*SOX2*	SRY (sex determining region Y)-box 2
Hs.195710	−2.08	*ZNF503*	zinc finger protein 503
Hs.535499	−2.02	*RARA*	retinoic acid receptor, alpha
Hs.310640	−2.02	*T2BP*	TRAF2-binding protein
Hs.513645	−2.02	*PAK6*	p21(CDKN1A)-activated kinase 6
Hs.131494	−2.00	*ARNT*	aryl hydrocarbon receptor nuclear translocator

Only genes with known protein products are shown.

a Genes annotations are from NCBI (http://www.ncbi.nlm.nih.gov).

b Fold changes are the average of four individuals.

**Table 3:** Genes up-regulated by ZnSO_4_

Gene accession no.^a^	Fold change^b^	Gene symbol^a^	Gene name^a^
Hs.188518	81.01	*MT1K*	metallothionein 1K
Hs.433391	28.87	*MT1G*	metallothionein 1G
Hs.283678	8.40	*PCDHB14*	protocadherin beta 14
Hs.412196	8.09	*ESRRBL1*	estrogen-related receptor beta-like 1
Hs.502182	5.46	*BDNF*	brain-derived neurotrophic factor
Hs.517581	4.78	*HMOX1*	heme oxygenase (decycling) 1
Hs.165736	4.67	*SCAND2*	SCAN domain containing 2
Hs.519469	4.65	*SLC30A1*	solute carrier family 30 (zinc transporter), member 1
Hs.513626	4.58	*MT1F*	metallothionein 1F (functional)
Hs.154296	4.58	*TLL2*	tolloid-like 2
Hs.303090	3.94	*PPP1R3C*	protein phosphatase 1, regulatory (inhibitor) subunit 3C
Hs.118354	3.66	*PRR3*	proline-rich polypeptide 3
Hs.466891	3.55	*ZNF233*	zinc finger protein 233
Hs.59889	3.47	*HMGCS2*	3-hydroxy-3-methylglutaryl-coenzyme A synthase 2 (mitochondrial)
Hs.278973	3.33	*ANGPT4*	angiopoietin 4
Hs.73962	3.31	*EPHA7*	EphA7
Hs.445835	3.22	*SERTAD4*	SERTA domain containing 4
Hs.352241	3.09	*TAS2R40*	taste receptor, type 2, member 40
Hs.78036	3.08	*SLC6A2*	solute carrier family 6 (neurotransmitter transporter, noradrenalin), member 2
Hs.89714	3.05	*CXCL5*	chemokine (C-X-C motif) ligand 5
Hs.195471	3.02	*PFKFB3*	6-phosphofructo-2-kinase/fructose-2,6-biphosphatase 3
Hs.460260	3.02	*AKR1C2*	aldoketo reductase family 1, member C2
Hs.16064	2.98	*MAGI1*	membrane-associated guanylate kinase interacting protein-like 1
Hs.143036	2.81	*CABP4*	calcium-binding protein 4
Hs.488671	2.67	*BAZ1B*	bromodomain adjacent to zinc finger domain, 1B
Hs.444450	2.62	*EGLN1*	egl nine homolog 1 (*C. elegans*)
Hs.465642	2.59	*SEMA6B*	sema domain, transmembrane domain (TM), and cyto plasmic domain, (semaphorin) 6B
Hs.32374	2.57	*DTX3*	deltex 3 homolog (*Drosophila*)
Hs.405667	2.49	*CD8B1*	CD8 antigen, beta polypeptide 1 (p37)
Hs.516664	2.48	*EFNA1*	ephrin-A1
Hs.487188	2.46	*MLLT4*	myeloid/lymphoid or mixed-lineage leukemia (trithorax homolog, *Drosophila*); translocated to, 4
Hs.6638	2.33	*MYEF2*	myelin expression factor 2
Hs.150136	2.25	*MAPK7*	mitogen-activated protein kinase 7
Hs.372000	2.24	*NSMAF*	neutral sphingomyelinase (N-SMase) activation associated factor
Hs.194721	2.21	*NCR2*	natural cytotoxicity triggering receptor 2
Hs.508720	2.19	*RAB20*	RAB20, member RAS oncogene family
Hs.522610	2.18	*LOC119180*	lysozyme 2
Hs.75535	2.16	*FOXN4*	forkhead box N4
Hs.485572	2.11	*SOCS2*	suppressor of cytokine signaling 2
Hs.521171	2.09	*HIG2*	hypoxia-inducible protein 2
Hs.80288	2.05	*HSPA1L*	heat-shock 70 kDa protein 1-like
Hs.123450	2.03	*JPH3*	junctophilin 3
Hs.441047	2.01	*ADM*	adrenomedullin

Only genes with known protein products are shown.

a Gene annotations are from NCBI (http://www.ncbi.nlm.nih.gov).

b Fold changes are the average of four individuals.

**Table 4:** Genes down-regulated by ZnSO_4_.

Gene accession no.^a^	Fold change^b^	Gene symbol^a^	Gene name^a^
Hs.376873	−6.25	*ZNF390*	zinc finger protein 390
Hs.106513	−6.09	*TLL1*	tolloid-like 1
Hs.200929	−5.87	*IL23R*	interleukin-23 receptor
Hs.268581	−5.47	*LPIN2*	lipin 2
Hs.112218	−5.36	*CAPN10*	calpain 10
Hs.532082	−5.23	*IL6ST*	interleukin 6 signal transducer (gp130, oncostatin M receptor)
Hs.483136	−4.53	*COMMD10*	COMM domain containing 10
Hs.141308	−4.39	*MOG*	myelin oligodendrocyte glycoprotein
Hs.7138	−4.10	*CHRM3*	cholinergic receptor, muscarinic 3
Hs.120633	−4.08	*SESN3*	sestrin 3
Hs.512587	−3.58	*MST1*	macrophage stimulating 1 (hepatocyte growth factor-like)
Hs.370510	−3.23	*IGSF4*	immunoglobulin superfamily, member 4
Hs.533040	−3.21	*PDLIM7*	PDZ and LIM domain 7 (enigma)
Hs.552578	−3.03	*TCF1*	transcription factor 1, hepatic; LF-B1, hepatic nuclear factor (HNF1), albumin proximal factor
Hs.472558	−2.92	*SDBCAG84*	serologically defined breast cancer antigen 84
Hs.506394	−2.77		ubiquitin specific protease 44
Hs.438994	−2.69	*ZNF544*	zinc finger protein 544
Hs.32721	−2.61	*SAG*	S-antigen; retina and pineal gland (arrestin)
Hs.74082	−2.48	*KLRC3*	killer cell lectin-like receptor subfamily C, member 3
Hs.382683	−2.47	*PRG-3*	plasticity-related gene 3
Hs.522291	−2.42	*PRKWNK2*	protein kinase, lysine deficient 2
Hs.493275	−2.34	*TRIM31*	tripartite motif-containing 31
Hs.129895	−2.29	*TBX3*	T-box 3 (ulnar mammary syndrome)
Hs.546263	−2.29	*KIR3DL2*	killer cell immunoglobulin-like receptor, three domains, long cytoplasmic tail, 2
Hs.546354	−2.21	*RRP4*	homolog of yeast RRP4 (ribosomal RNA processing 4), 3′-5′-exoribonuclease
Hs.19385	−2.17	*ABHD5*	abhydrolase domain containing 5
Hs.344400	−2.19	*MPHOSPH6*	M-phase phosphoprotein 6
Hs.411311	−2.17	*IL24*	interleukin 24
Hs.492236	−2.17	*H326*	H326
Hs.255432	−2.06	*CIB3*	calcium and integrin binding family member 3
Hs.476052	−2.02	*SNRK*	SNF-1 related kinase
Hs.432898	−2.01	*MAP3K13*	mitogen-activated protein kinase kinase kinase 13

Only genes with known protein products are shown.

a Gene annotations are from NCBI (http://www.ncbi.nlm.nih.gov).

b Fold changes are the average of four individuals.

## References

[b1-ehp0113-001747] Affymetrix, Inc 2004a. Expression Analysis Technical Manual. Available: http://www.affymetrix.com/support/technical/manual/expression_manual/affx [accessed 20 October 2005]

[b2-ehp0113-001747] Affymetrix, Inc 2004b. Statistical Algorithms Reference Guide. Available: http://www.affymetrix.com/support/technical/technotesmain.affx [accessed 20 October 2005].

[b3-ehp0113-001747] Andrew AS, Warren AJ, Barchowsky A, Temple KA, Klei L, Soucy NV (2003). Genomic and proteomic profiling of responses to toxic metals in human lung cells. Environ Health Perspect.

[b4-ehp0113-001747] Arend WP (2002). The balance between IL-1 and IL-1Ra in disease. Cytokine Growth Factor Rev.

[b5-ehp0113-001747] Bajalica-Lagercrantz S, Piehl F, Farnebo F, Larsson C, Lagercrantz J (1998). Expression of the *BCL6* gene in the pre-and postnatal mouse. Biochem Biophys Res Commun.

[b6-ehp0113-001747] Becker S, Quay J, Koren HS, Haskill JS (1994). Constitutive and stimulated MCP-1, *GRO* alpha, beta, and gamma expression in human airway epithelium and bronchoalveolar macrophages. Am J Physiol.

[b7-ehp0113-001747] Bonner JC, Lindroos PM, Rice AB, Moomaw CR, Morgan DL (1998). Induction of PDGF receptor-alpha in rat myofibroblasts during pulmonary fibrogenesis *in vivo*. Am J Physiol.

[b8-ehp0113-001747] Bonner JC, Rice AB, Moomaw CR, Morgan DL (2000). Airway fibrosis in rats induced by vanadium pentoxide. Am J Physiol Lung Cell Mol Physiol.

[b9-ehp0113-001747] Boucher CA, Carey N, Edwards YH, Siciliano MJ, Johnson KJ (1996). Cloning of the human *SIX1* gene and its assignment to chromosome 14. Genomics.

[b10-ehp0113-001747] Bray P, Lichter P, Thiesen HJ, Ward DC, Dawid IB (1991). Characterization and mapping of human genes encoding zinc finger proteins. Proc Natl Acad Sci USA.

[b11-ehp0113-001747] Cadene A, Grigorescu F, Serrano JJ, Cros G (1997). Characterization of vanadyl sulfate effect on vascular contraction: roles of calcium and tyrosine phosphorylation. J Pharmacol Exp Ther.

[b12-ehp0113-001747] Calderon-Garciduenas L, Maronpot RR, Torres-Jardon R, Henriquez-Roldan C, Schoonhoven R, Acuna-Ayala H (2003). DNA damage in nasal and brain tissues of canines exposed to air pollutants is associated with evidence of chronic brain inflammation and neurodegeneration. Toxicol Pathol.

[b13-ehp0113-001747] Carter JD, Ghio AJ, Samet JM, Devlin RB (1997). Cytokine production by human airway epithelial cells after exposure to an air pollution particle is metal-dependent. Toxicol Appl Pharmacol.

[b14-ehp0113-001747] Chen F, Vallyathan V, Castranova V, Shi X (2001). Cell apoptosis induced by carcinogenic metals. Mol Cell Biochem.

[b15-ehp0113-001747] Conrad CC, Walter CA, Richardson A, Hanes MA, Grabowski DT (1997). Cadmium toxicity and distribution in metallothionein-I and -II deficient transgenic mice. J Toxicol Environ Health.

[b16-ehp0113-001747] Cosma G, Fulton H, DeFeo T, Gordon T (1992). Rat lung metallothionein and heme oxygenase gene expression following ozone and zinc oxide exposure. Toxicol Appl Pharmacol.

[b17-ehp0113-001747] Courtade M, Carrera G, Paternain JL, Martel S, Carre PC, Folch J (1998). Metallothionein expression in human lung and its varying levels after lung transplantation. Toulouse Lung Transplantation Group. Chest.

[b18-ehp0113-001747] Doig AT, Challen PJ (1964). Respiratory hazards in welding. Ann Occup Hyg.

[b19-ehp0113-001747] Evans EH (1945). Casualties following exposure to zinc chloride smoke. Lancet.

[b20-ehp0113-001747] Flenghi L, Fagioli M, Tomassoni L, Pileri S, Gambacorta M, Pacini R (1995). Characterization of a new monoclonal antibody (PG-M3) directed against the aminoterminal portion of the PML gene product: immunocytochemical evidence for high expression of PML proteins on activated macrophages, endothelial cells, and epithelia. Blood.

[b21-ehp0113-001747] Gavett SH, Madison SL, Dreher KL, Winsett DW, McGee JK, Costa DL (1997). Metal and sulfate composition of residual oil fly ash determines airway hyperreactivity and lung injury in rats. Environ Res.

[b22-ehp0113-001747] Gavett SH, Madison SL, Stevens MA, Costa DL (1999). Residual oil fly ash amplifies allergic cytokines, airway responsiveness, and inflammation in mice. Am J Respir Crit Care Med.

[b23-ehp0113-001747] Ghio AJ, Kim C, Devlin RB (2000). Concentrated ambient air particles induce mild pulmonary inflammation in healthy human volunteers. Am J Respir Crit Care Med.

[b24-ehp0113-001747] Gon Y, Asai Y, Hashimoto S, Mizumura K, Jibiki I, Machino T (2004). A20 inhibits toll-like receptor 2- and 4-mediated inter-leukin-8 synthesis in airway epithelial cells. Am J Respir Cell Mol Biol.

[b25-ehp0113-001747] Haskill S, Peace A, Morris J, Sporn SA, Anisowicz A, Lee SW (1990). Identification of three related human *GRO* genes encoding cytokine functions. Proc Natl Acad Sci USA.

[b26-ehp0113-001747] He KL, Ting AT (2002). A20 inhibits tumor necrosis factor (TNF) alpha-induced apoptosis by disrupting recruitment of TRADD and RIP to the TNF receptor 1 complex in Jurkat T cells. Mol Cell Biol.

[b27-ehp0113-001747] Huang C, Zhang Z, Ding M, Li J, Ye J, Leonard SS (2000). Vanadate induces p53 transactivation through hydrogen peroxide and causes apoptosis. J Biol Chem.

[b28-ehp0113-001747] Huang YC, Ghio AJ, Stonehuerner J, McGee J, Carter JD, Grambow SC (2003). The role of soluble components in ambient fine particles-induced changes in human lungs and blood. Inhal Toxicol.

[b29-ehp0113-001747] Huang YC, Wu W, Ghio AJ, Carter JD, Silbajoris R, Devlin RB (2002). Activation of EGF receptors mediates pulmonary vasoconstriction induced by residual oil fly ash. Exp Lung Res.

[b30-ehp0113-001747] Ingram JL, Rice AB, Santos J, Van Houten B, Bonner JC (2003). Vanadium-induced HB-EGF expression in human lung fibroblasts is oxidant dependent and requires MAP kinases. Am J Physiol Lung Cell Mol Physiol.

[b31-ehp0113-001747] Irato P, Santovito G, Piccinni E, Albergoni V (2001). Oxidative burst and metallothionein as a scavenger in macrophages. Immunol Cell Biol.

[b32-ehp0113-001747] Johnson MC, Kajikawa O, Goodman RB, Wong VA, Mongovin SM, Wong WB (1996). Molecular expression of the alpha-chemokine rabbit *GRO* in *Escherichia coli* and characterization of its production by lung cells *in vitro* and *in vivo*. J Biol Chem.

[b33-ehp0113-001747] Kagi JHR 1993. Evolution, structure and chemical activity of class I metallothioneins: an overview. In: Metallothionein III (Suzuki KT, Imura N, Kimura M, eds). Basel:Birkhauser Verlag, 29–55.

[b34-ehp0113-001747] Karin M (1985). Metallothioneins: proteins in search of function. Cell.

[b35-ehp0113-001747] Kuschner WG, D’Alessandro A, Wintermeyer SF, Wong H, Boushey HA, Blanc PD (1995). Pulmonary responses to purified zinc oxide fume. J Investig Med.

[b36-ehp0113-001747] Laclef C, Hamard G, Demignon J, Souil E, Houbron C, Maire P (2003). Altered myogenesis in Six1-deficient mice. Development.

[b37-ehp0113-001747] Levy BS, Hoffman L, Gottsegen S (1984). Boilermakers’ bronchitis. Respiratory tract irritation associated with vanadium pentoxide exposure during oil-to-coal conversion of a power plant. J Occup Med.

[b38-ehp0113-001747] Magari SR, Schwartz J, Williams PL, Hauser R, Smith TJ, Christiani DC (2002). The association of particulate air metal concentrations with heart rate variability. Environ Health Perspect.

[b39-ehp0113-001747] Marquart H, Smid T, Heederik D, Visschers M (1989). Lung function of welders of zinc-coated mild steel: cross-sectional analysis and changes over five consecutive work shifts. Am J Ind Med.

[b40-ehp0113-001747] Matarese SL, Matthews JI (1986). Zinc chloride (smoke bomb) inhalational lung injury. Chest.

[b41-ehp0113-001747] McDowell SA, Gammon K, Bachurski CJ, Wiest JS, Leikauf JE, Prows DR (2000). Differential gene expression in the initiation and progression of nickel-induced acute lung injury. Am J Respir Cell Mol Biol.

[b42-ehp0113-001747] Mukherjee B, Patra B, Mahapatra S, Banerjee P, Tiwari A, Chatterjee M (2004). Vanadium—an element of atypical biological significance. Toxicol Lett.

[b43-ehp0113-001747] Nadadur SS, Schladweiler MC, Kodavanti UP (2000). A pulmonary rat gene array for screening altered expression profiles in air pollutant-induced lung injury. Inhal Toxicol.

[b44-ehp0113-001747] Nemery B (1990). Metal toxicity and the respiratory tract. Eur Respir J.

[b45-ehp0113-001747] Nriagu JO, Pacyna JM (1988). Quantitative assessment of worldwide contamination of air, water and soils by trace metals. Nature.

[b46-ehp0113-001747] Ohno H (2004). Pathogenetic role of BCL6 translocation in B-cell non-Hodgkin’s lymphoma. Histol Histopathol.

[b47-ehp0113-001747] Pare CM, Sandler M (1954). Smoke-bomb pneumonitis: description of a case. J R Army Med Corps.

[b48-ehp0113-001747] Park JD, Liu Y, Klaassen CD (2001). Protective effect of metallothionein against the toxicity of cadmium and other metals(1). Toxicology.

[b49-ehp0113-001747] Rangnekar VV, Waheed S, Davies TJ, Toback FG, Rangnekar VM (1991). Antimitogenic and mitogenic actions of interleukin-1 in diverse cell types are associated with induction of *gro* gene expression. J Biol Chem.

[b50-ehp0113-001747] Riley MR, Boesewetter DE, Kim AM, Sirvent FP (2003). Effects of metals Cu, Fe, Ni, V, and Zn on rat lung epithelial cells. Toxicology.

[b51-ehp0113-001747] Samet JM, Graves LM, Quay J, Dailey LA, Devlin RB, Ghio AJ (1998). Activation of MAPKs in human bronchial epithelial cells exposed to metals. Am J Physiol.

[b52-ehp0113-001747] Samet JM, Silbajoris R, Wu W, Graves LM (1999). Tyrosine phosphatases as targets in metal-induced signaling in human airway epithelial cells. Am J Respir Cell Mol Biol.

[b53-ehp0113-001747] Sato H, Sagai M, Suzuki KT, Aoki Y (1999). Identification, by cDNA microarray, of A-raf and proliferating cell nuclear antigen as genes induced in rat lung by exposure to diesel exhaust. Res Commun Mol Pathol Pharmacol.

[b54-ehp0113-001747] Walsh CT, Sandstead HH, Prasad AS, Newberne PM, Fraker PJ (1994). Zinc: health effects and research priorities for the 1990s. Environ Health Perspect.

[b55-ehp0113-001747] Wertz IE, O’Rourke KM, Zhou H, Eby M, Aravind L, Seshagiri S (2004). Deubiquitination and ubiquitin ligase domains of A20 downregulate NF-kappaB signalling. Nature.

[b56-ehp0113-001747] Woodin MA, Hauser R, Liu Y, Smith TJ, Siegel PD, Lewis DM (1998). Molecular markers of acute upper airway inflammation in workers exposed to fuel-oil ash. Am J Respir Crit Care Med.

[b57-ehp0113-001747] Woodin MA, Liu Y, Hauser R, Smith TJ, Christiani DC (1999). Pulmonary function in workers exposed to low levels of fuel-oil ash. J Occup Environ Med.

[b58-ehp0113-001747] Woodin MA, Liu Y, Neuberg D, Hauser R, Smith TJ, Christiani DC (2000). Acute respiratory symptoms in workers exposed to vanadium-rich fuel-oil ash. Am J Ind Med.

[b59-ehp0113-001747] Wu W, Graves LM, Jaspers I, Devlin RB, Reed W, Samet JM (1999). Activation of the EGF receptor signaling pathway in human airway epithelial cells exposed to metals. Am J Physiol.

[b60-ehp0113-001747] Wu W, Jaspers I, Zhang W, Graves LM, Samet JM (2002). Role of Ras in metal-induced EGF receptor signaling and NF-kappaB activation in human airway epithelial cells. Am J Physiol Lung Cell Mol Physiol.

[b61-ehp0113-001747] Xu PX, Zheng W, Huang L, Maire P, Laclef C, Silvius D (2003). Six1 is required for the early organogenesis of mammalian kidney. Development.

[b62-ehp0113-001747] Zhang L, Rice AB, Adler K, Sannes P, Martin L, Gladwell W (2001). Vanadium stimulates human bronchial epithelial cells to produce heparin-binding epidermal growth factor-like growth factor: a mitogen for lung fibroblasts. Am J Respir Cell Mol Biol.

[b63-ehp0113-001747] Zhong S, Salomoni P, Pandolfi PP (2000). The transcriptional role of *PML* and the nuclear body. Nat Cell Biol.

